# Comparative study of the anti-inflammatory effect and acute toxicity of Sacha Inchi oils (*Plukenetia volubilis* and *Plukenetia huayllabambana*) in mice

**DOI:** 10.3389/fimmu.2025.1641344

**Published:** 2025-10-13

**Authors:** Jose Luis Aguilar-Olano, Silvia Davila Paico, Christian Pitot Alvarez

**Affiliations:** ^1^ Laboratorio de Inmunología, Departamento de Ciencias Celulares y Moleculares, Facultad de Ciencias e Ingeniería, Universidad Peruana Cayetano Heredia, Lima, Peru; ^2^ Facultad de Medicina Veterinaria y Zootecnia, Universidad Peruana Cayetano Heredia, Lima, Peru

**Keywords:** *Plukenetia volubilis*, *Plukenetia huayabambana*, anti-inflammatory, omega-3, cytotoxicity, Sacha inchi

## Abstract

**Introduction:**

The seed of Sacha Inchi (*Plukenetia* spp.), a plant native from the Peruvian Amazon, has a high content of Omega 3 (ω-3) in its composition, reaching up to 53% of its total fatty acids. Fatty acids ω-3 can significantly reduce the production of proinflammatory molecules and could therefore inhibit or reduce the inflammatory response.

**Objective:**

We evaluate the oils from the seeds of two species of Sacha Inchi: *Plukenetia volubilis* and *Plukenetia huayllabambana* for their inflammation inhibitory capacity, in an *in vivo* and *in vitro* model in experimental animals.

**Results:**

The physicochemical analysis showed that Sacha Inchi oil obtained from *P. huayllabambana* seeds contained 53% ω-3, whereas that obtained from *P. volubilis* presented 47%. The evaluation of cytotoxicity in culture of normal splenocytes demonstrated that both oils are non-cytotoxic, because they showed a median inhibitory concentration (IC50) of 48,561 mg/mL for *P. volubilis* and 50,695 mg/mL for *P. huayllabambana*, which, physiologically, are values impossible to achieve in cells. In the evaluation of acute toxicity (mean lethal dose, LD50) in C57 mice, *P. volubilis* obtained the value of 63,603 mg/kg, whereas *P. huayllabambana* obtained 74,638 mg/kg, which classifies both oils in the category of Relatively Harmless, that is, non-toxic for the models tested. Both oils demonstrated a statistically significant anti-inflammatory effect, with *P. huayllabambana* oil being the most effective, presenting a 62% percentage of inhibition of inflammation. We demonstrate the safety and potential anti-inflammatory activity of *Plukenetia* sp., which could serve as a basis for future research.

## 1 Introduction

Sacha Inchi, *Plukenetia* spp., corresponds to two members of the genus *Plukenetia* existing in Peru: *Plukenetia volubilis* and *Plukenetia huayllabambana*. These are oilseed, climbing plants native to the Peruvian Amazon (1).

These seeds have a high protein (33%) and oil (49%) (2) content, as well as other nutrients. It contains α-linolenic omega 3 (ALA ω-3) fatty acids between 45% and 54%, linoleic omega 6 acid (LA ω-6) at 36%, and oleic omega 9 acid (OA w-9) at 9% (2, 3). As a comparison, fish oil contains between 0.1% and 5.2% of ALA ω-3, but 10% to 20% eicosapentaenoico (EPA ω-3), 8% to 15% docosahexaenoic acid (DHA ω-3), 1% to 4% of ω-6, and 10% to 25% of ω-9 (4).

Both ω-3 and ω-6 fatty acids are essential polyunsaturated fatty acids. The enzymes (Δ12-desaturase and Δ15-desaturase) are found only in plants; therefore, vegetable tissues and oils, or animals that consume them, are a rich source of omega fatty acids (5).

Linoleic fatty acid is in high concentrations in soybean, corn, safflower, and sunflower oils, whereas ALA ω-3 is present in some fish such as tuna, sardine, trout, mackerel, and horse mackerel; in green leafy vegetables; and in olive, linseed, walnut, etc., oils (6).

The same enzymes metabolize both ω-6 and ω-3. Both compete with each other through the same metabolic pathway, however, giving rise to different molecules. Arachidonic acid (ARA) derives from ω-6, and EPA and DHA from ω-3. These fatty acids are mainly in cell membranes. In addition, ARA, EPA, and DHA are precursors of the final metabolites called eicosanoids (prostaglandins, thromboxanes, and leukotrienes) (5).

Eicosanoids from ARA produce metabolites of series 2: prostaglandin E2, thromboxane A2, and prostacyclin I2; and leukotrienes of series 4: A4, B4, C4, and D4. These are pro-inflammatory: capillary vasodilators, chemotactic for polymorphonuclear cells (PMN) and macrophages, and prothrombotics, whereas those from EPA and DHA produce metabolites of series 3: prostaglandin E3, thromboxane A3, prostacyclin I3; leukotrienes of series 5—A5, B5, C5, and D5—are the complete opposite: anti-inflammatory, antithrombotic, vasoconstrictors, antiarrhythmic, block PMN, and macrophage chemotactic (5).

In addition, ω-3 metabolites will produce several types of resolvins, protectins, and maresins, which will fulfill tissue repair functions. After an inflammatory process induced by a pathogen attack on a tissue, there will be areas of tissue damage (endothelial, collagen fibers, and fibroblasts) that will require repair when the infection is controlled. These repair lipid molecules from ω-3 actively participate in this process (5, 6).

Given the scarce availability of ω-3, and growing population in cities, there is a decrease in the intake of ω-3 fatty acids in humans (5–8). If in our diet we ingest a high content of ω-6, but low ω-3, the content of ARA in our cells will be much higher than that of EPA and DHA. Then, we can mount an adequate inflammatory defense response, but then there will be difficulty in activating anti-inflammatory regulatory mechanisms, leading to chronic inflammatory conditions (5, 10).

**Figure 1:**
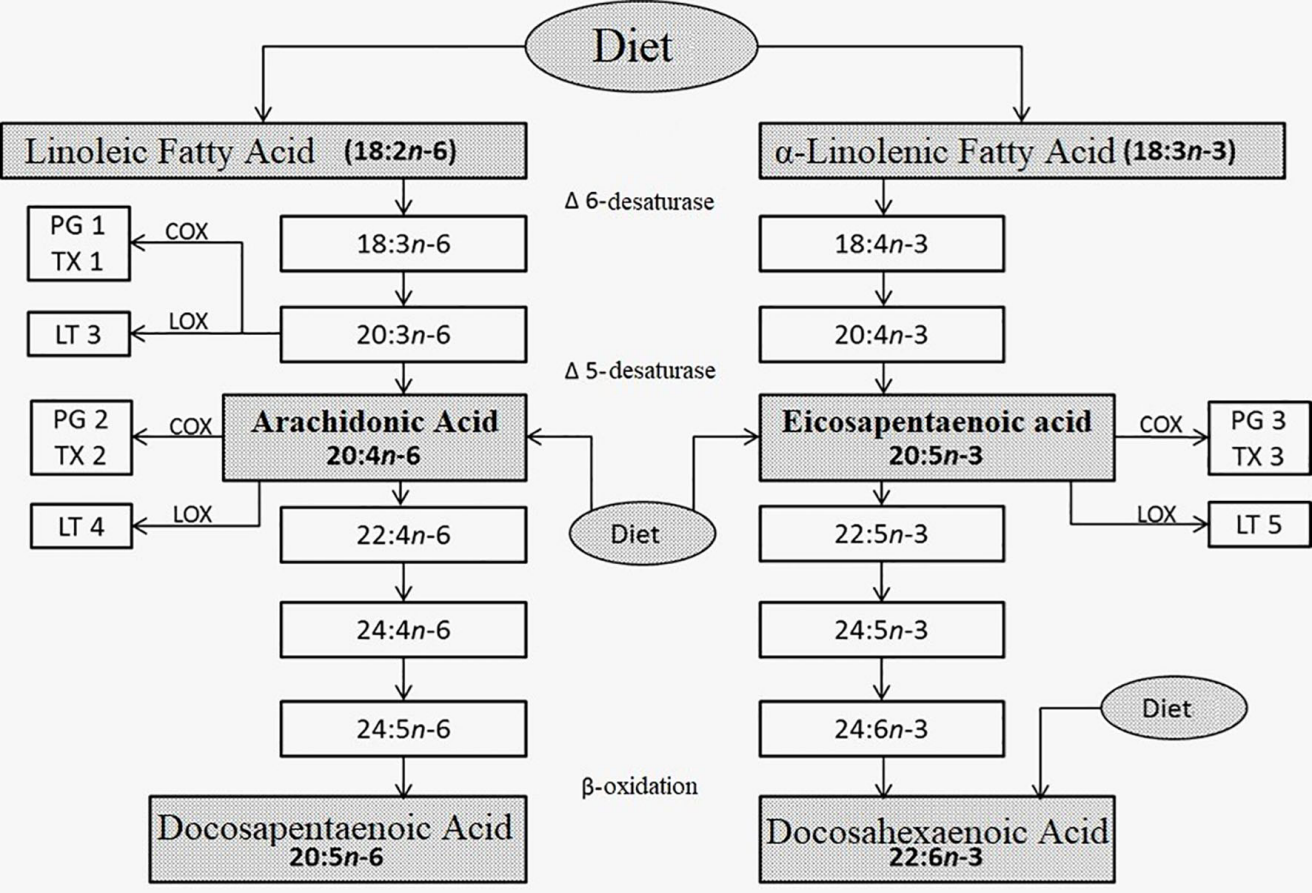
Metabolism of linoleic (left) and α-linolenic (right) fatty acids. Taken, translated, and modified from References 5-9.

Eicosanoids (**Figure 1**) are involved in the regulation of the intensity and duration of inflammation, and in other effector mechanisms of the immune response. Inflammation requires careful regulation to avoid the development of chronic inflammatory diseases, such as autoimmune diseases, allergies, cancer, arteriosclerosis, or Alzheimer’s (11–16).

Various publications support the anti-inflammatory mechanisms of fatty acid derivatives. The resolvin E1 molecule, which derives from ω-3, would interact with leukotriene B4, receptor BLT1, and ChemR23, attenuate the activation of the NF-κB transcription factor, and then regulate inflammation (11, 13, 15, 16).

Sacha Inchi oil, a rich source of ALA 1ω-3, becomes a good alternative for study.

We investigated two varieties of Sacha Inchi oil, extracted from *P. huayllabambana* and *P. volubilis*. The LD_50_ dose was established in mice and the mean inhibitory concentration in cell cultures (IC_50_) of both varieties, and their anti-inflammatory capacity was demonstrated *in vivo* and *in vitro* in an animal model of inflammation induced by λ-carrageenan (17, 18).

## 2 Materials and methods

### 2.1 About used Sacha Inchi oils

We used Extra Virgin oil of Sacha Inchi (*Plukenetia*). The seeds of *P. volubilis* were harvested in the low jungle of the department of San Martín, Peru, while those of *P. huayllabambana* were collected from the province of Rodríguez de Mendoza (high jungle), located in the department of Amazonas, Peru. Both types of seeds were 100% organic (grown without pesticides or fertilizers) and cold pressed.

### 2.2 Physicochemical analysis of Sacha Inchi oil

#### 2.2.1 Relative density (ρ)

The determination of the density of the oil was carried out at a standard temperature of 22°C using a 10-mL glass pycnometer and a 4-digit Adventurer OHAUS analytical balance.

#### 2.2.2 Composition of saturated and unsaturated fatty acids

The fatty acid composition was determined by the methylation derivatization method. Briefly, 100 mL of oil was dissolved in 10 mL of pentane. KOH/methanolic (2M) was then added and vortexed (Vortex IKA – MS3 Basic) for 2 min at room temperature. The supernatant was passed through a Pasteur pipette that had a cotton layer and collected in an amber vial for analysis in the Gas Chromatograph (Agilent Technologies 7890A) coupled to a Mass Spectrometer (Agilent Technologies 5975C).

The carrier gas was helium (purity > 99.99%), and the DB–FFAP capillary column (0.25 mm × 60 m × 0.25 µm) (J&W Scientific) was used. The column temperature was maintained at 165°C for 4 min and then increased by 4°C/min to 185°C, where it was held for 5 min, and then increased at 10°C/min until the final temperature of 225°C was reached and maintained for 10 min. The sample injection volume was 1 μL. The identification of the components was based on two criteria:

1. Comparison of the retention time and mass spectra of a mixture of fatty acid methyl ester standards (Supelco 37 component FAME Mix, PA, USA);
2. Comparison of their mass spectra with the NIST 08 and FLAVOR 02 spectral libraries.

### 2.3 Acute toxicity evaluation (mean lethal dose, LD_50_)

Male and female strain C57 mice were used, obtained from the UPCH vivarium, with body weights between 22 and 32 g, in an age range between 8 and 10 weeks. The mice were at room temperature and on a light/dark cycle of 12 h each. The feeding was based on a balanced diet for vivarium mice and water *ad libitum*.

Six groups of six mice each were formed. Prior to the administration of the products, the animals had a 24-h period of fasting and adaptation. The first group was administered 10 mL/kg, the second 17.4 mL/kg, the third 30.2 mL/kg, the fourth 52.5 mL/kg, the fifth 91.2 mL/kg, and the sixth 150 mL/kg of each of the oils or as control group; vegetable cooking oil (CIL brand, batch: 1623L) was administered.

The highest and lowest doses were established according to the results obtained in the pilot trials. The other doses were determined using a logarithmic ratio (17, 18).

In all cases, the oil was administered orally through a No. 20 orogastric cannula. The animals were constantly observed during the first 24 h and once a day until 14 days after the administration of the oils, recording any observable changes. Likewise, we monitored body weight daily.

After this period, the animals were euthanized with Halatal^®^ (pentobarbital) at an intraperitoneal dose of 100 mg/kg of body weight. The sacrificed surviving animals and any mice that died during the experiment all underwent necropsy to rule out any visceral damage. Samples were taken from the lung, heart, spleen, thymus, stomach, liver, kidney, pancreas, and brain and fixed in formalin (4%) until processed for histological analysis, using the hematoxylin–eosin (HE) staining technique.

The study was approved by the UPCH Institutional Animal Ethics Committee, with code No. SIDISI 56965.

The LD_50_ value was determined using the Probits statistical method (EPA Probit Analysis Program) (19).

### 2.4 Evaluation of cytotoxicity (IC_50_)

The determination of the IC_50_ for *P. volubilis* and *P. huayllabambana* was performed by isolating spleen cells (splenocytes) from healthy untreated C57/BL6 mice. For this test, three spleens from different mice were used. Splenocytes extracted were cultured in triplicate for each concentration of Sacha Inchi used, including the negative control.

Because Sacha Inchi oil is not soluble in water, we solubilize in β-cyclodextrin (Sigma^®^) at 0.013%, which together with the diluent (in this case RPMI - 1640 [Roswell Park Memorial Institute Medium]) forms an aggregate capable of stabilizing the oil in aqueous solution. This compound is used in cell cultures and does not interfere with the purposes of the assay (20).

Splenocytes were subjected to different concentrations of Sacha Inchi. For both the *P. volubilis* and *P. huayllabambana* varieties, serial dilutions were used, from 100% (which was pure Sacha Inchi oil) to 3.13%. Cells were cultured for 48 and 72 h at 37°C and 5% CO_2_.

The determination of IC_50_ was carried out using the MTT (21) test, a technique already standardized in our laboratory. Briefly, after 3 h at a 37°C incubation time, 15 µL of the MTT reagent (3-(4,5-diamethylthiazol-2-yl)-2,5-diphenyl tetrasodium bromide) was added to all culture wells and incubation continued for 4 more hours. Afterward, we added 150 µL of isopropanol–HCl to all wells to dissolve the formazan crystals. Then, we read the culture plate in a spectrophotometer (VersaMax ELISA microplate reader from Molecular Devices) at 550 nm with a reference filter at 630 nm. All measurements were made in triplicate, and the mean and standard error of the mean (SE) were obtained. To determine IC_50_, we used the GraphPad program, to perform the sigmoidal dose–response analysis.

### 2.5 Evaluation of the anti-inflammatory effect *in vivo* model

The experimental model of plantar edema induced by λ-carrageenan was used for the anti-inflammatory effect of the two varieties of Sacha Inchi oil (18).

The C57 males and females mice were acquired from the UPCH vivarium, with body weights between 18 and 22 g and ages between 6 and 8 weeks.

For both oils, four groups of eight mice each were used; the first group was administered 40 mL/kg of body weight per day; to the second group, 20 mL/kg/day; to the third 10 mL/kg/day; and to the last group, 5 mL/kg/day.

The mice received the respective oils through a No. 20 blunt-tip metal gastric tube for 7 days before causing inflammation. One hour after the administration of the last dose of the oil, 0.05 mL of 1% λ-carrageenan (Sigma^®^) diluted in saline solution was applied to the right plantar aponeurosis to induce inflammation, as described by Sugishita et al. (18)

There were three control groups. The first received distilled water as treatment during the 7 days, and the plantar aponeurosis received saline solution (negative control). The second control group received, through a gastric tube, 1 mL of indomethacin (Sigma^®^) at a concentration of 7 mg/kg (diluted in 0.1 M Na_2_CO_3_) as an anti-inflammatory agent (anti-inflammatory control), administered 1 h before the induction of inflammation, whereas the third group received distilled water as treatment (positive control). Both the second and third groups received 1% λ-carrageenan to the plantar aponeurosis.

Inflammation was measured using a micrometer, 2 and 4 h after administering λ-carrageenan. The difference in inflammation was found from the subtraction between the measurements of the inflamed paw (paw stimulated with λ-carrageenan) minus the measurement of the non-inflamed paw (control paw of the same individual).

The statistical calculations were carried out taking into account the measurements made 2 h after the induction of inflammation, based on the results obtained in the pilot trials. To find the difference between the experimental groups, the GraphPad program was used, with the non-parametric Mann–Whitney test. Furthermore, based on these data, we calculated the percentage of inhibition of the inflammatory reaction.

## 3 Results

### 3.1 Physicochemical analysis of Sacha Inchi oil

The physicochemical analysis had two tests: the relative density and the determination of the composition of saturated and unsaturated fatty acids, to determine the concentrations of the constituent compounds of both oils.

#### 3.1.1 Relative density (ρ)

The density of the oil was measured using a pycnometer and an analytical balance, as mentioned in Materials and Methods (see **Tables 1**, **2**).

**Table 1:** The weight values obtained for the determination of the density of the *P. volubilis oil*.

Repetitions	Empty pycnometer (g)	Pycnometer with water (g)	Pycnometer with oil (g)	Relative density (g/mL)
1	9.6791	12.1063	11.9293	0.92708
2	9.679	12.1061	11.9292	0.92711
3	9.6791	12.1061	11.9294	0.92719
			**Average (g/mL)**	**0.92713**

Relative density obtained on average (bold) from 3 repetitions of measurements on the pycnometer.

**Table 2:** The weight values obtained for the determination of the density of the *P. huayllabambana* oil.

Repetitions	Empty pycnometer (g)	Pycnometer with water (g)	Pycnometer with oil (g)	Relative density (g/mL)
1	9.6792	12.1062	11.9255	0.92555
2	9.6791	12.1063	11.9254	0.92547
3	9.679	12.1063	11.9255	0.92551
			**Average (g/mL)**	**0.92551**

Relative density obtained on average (bold) from 3 repetitions of measurements on the pycnometer.

#### 3.1.2 Composition of saturated and unsaturated fatty acids

The methylation derivatization method was used, taking into account two criteria: the comparison of the retention time and the mass spectra of a mixture of fatty acid methyl ester standards. Then, their mass spectra were compared with the NIST 08 and FLAVOR 02.17 spectral library.

**Figure 2:**
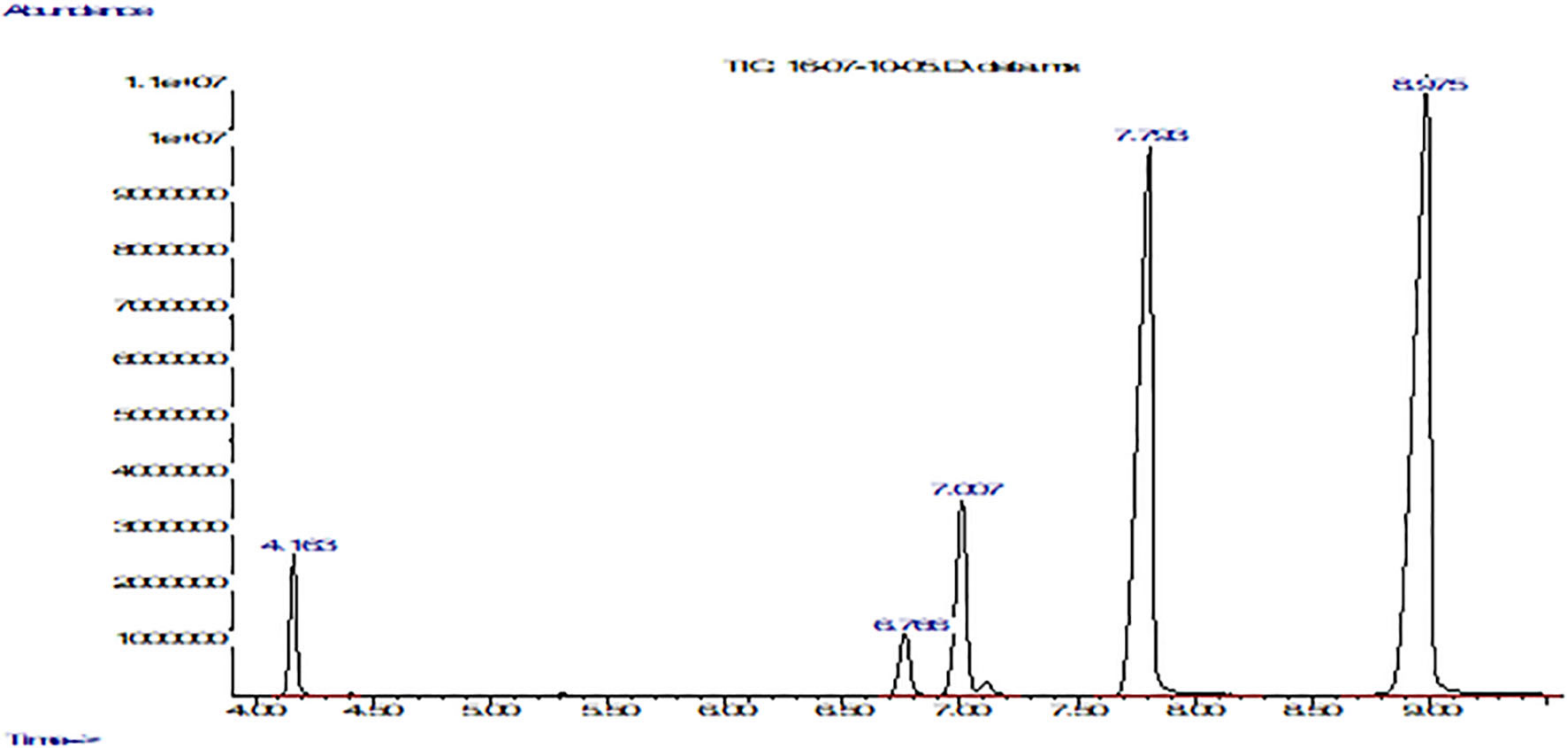
Fatty acid composition of *P. volubilis* with FFAP column using a gas chromatograph coupled to a mass spectrometer. The graph shows abundance vs. time (seconds). From left to right: the first peak corresponds to palmitic acid, the second to stearic acid, the third to oleic acid, the fourth peak to linoleic acid, and finally the fifth and last peak belongs to α-linolenic acid.

**Figure 3:**
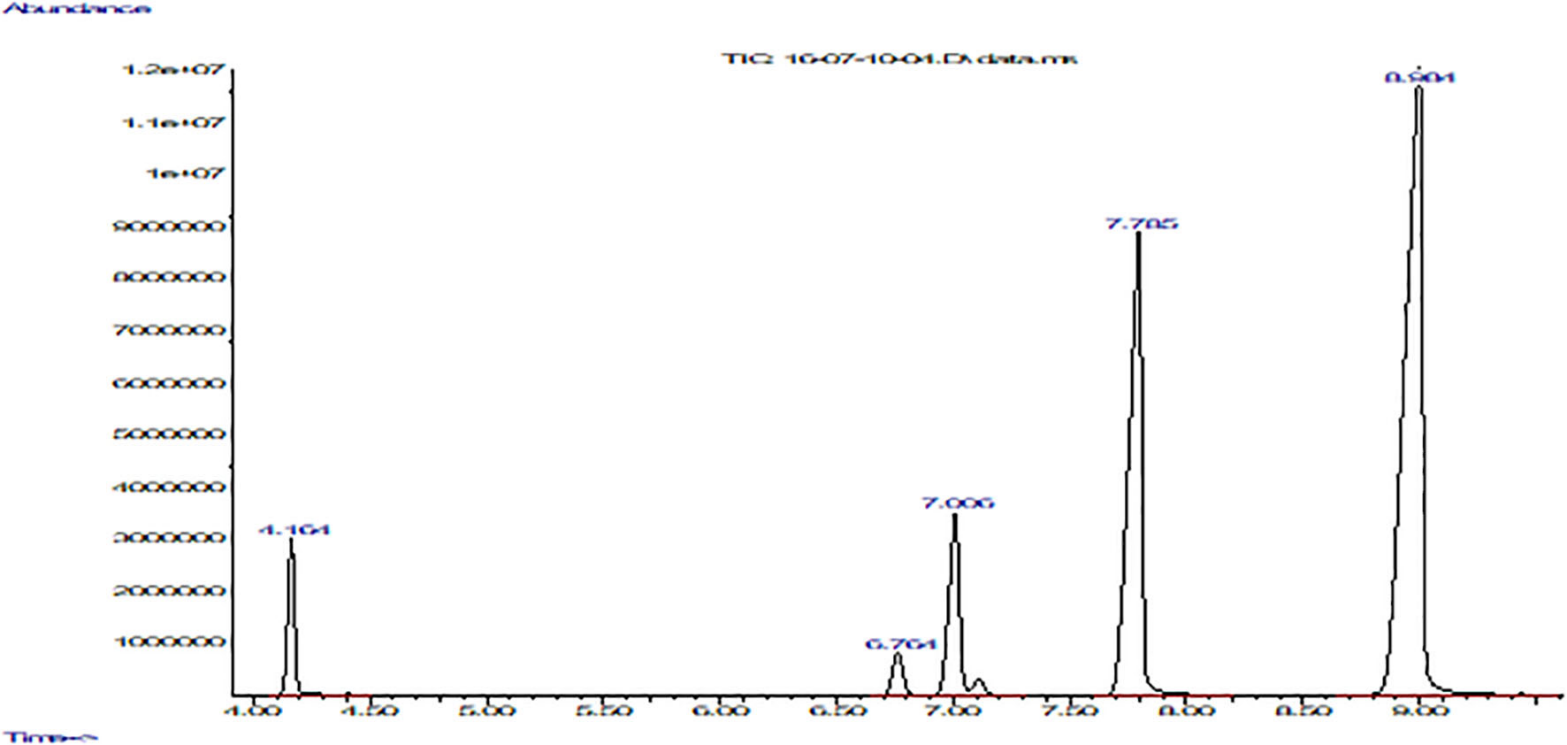
Fatty acid composition of *P. huayllabambana* with FFAP column using a gas chromatograph coupled to a mass spectrometer. The graph shows abundance vs. time (seconds). From left to right: the first peak corresponds to palmitic acid, the second to stearic acid, the third to oleic acid, the fourth peak to linoleic acid, and finally the fifth and last peak belongs to α-linolenic acid.

**Table 3** and **Figure 2** show the fatty acid composition of *P. volubilis* and **Table 4** and **Figure 3** for *P. huayllabambana*.

**Table 3:** Fatty acid composition for *P. volubilis*.

Fatty acid	Relative %
Palmitic (16:0)	4.46
Linoleic or Omega 6 (18:2)	35.53
α-Linolenic or Omega 3 (18:3)	46.65
Oleic (18:1)	10.32
Estearic (18:0)	3.04
Ratio Omega 3:Omega 6	1.31

**Table 4:** Fatty acid composition for *P. huayllabambana*.

Fatty acid	Relative %
Palmitic (16:0)	5.29
Linoleic u Omega 6 (18:2)	29.17
α-Linolenic u Omega 3 (18:3)	53.20
Oleic (18:1)	10.13
Estearic (18:0)	2.21
	
Ratio Omega 3:Omega 6	1.82

### 3.2 Assessment of acute toxicity (median lethal dose)

The mean lethal dose (LD_50_) included the evaluation of two aspects: the physiological and the morphological. The data were analyzed using the Probits statistical method using the EPA Probit Analysis Program software.

**Table 5** shows that there was mortality from group No. 4 (52.5 mL/kg). In addition, the animals showed sweating from group No. 3 onward, varying from mild to moderate, in a dose-dependent manner. This symptom appeared 2 h after the administration of the oil.

**Table 5:** LD_50_ data of mice treated with *P. volubilis* at 7 days after treatment.

Group	Number animals	Average weight of mice (g)	Doses (mL/kg)	Managed volume (mL)	No. of deaths	No. of living
N°1	6	25.33	10	0.25	0	6
N°2	6	26.83	17.378	0.47	0	6
N°3	6	27	30.1995	0.82	0	6
N°4	6	28	52.4807	1.47	1	5
N°5	6	28.33	91.2011	2.58	5	1
N°6	6	29	150	4.35	6	0
Cooking oil	6	30.5	150	4.58	0	6

After the fifth day, sweating decreased noticeably in the animals in groups Nos. 3, 4, and 5, until it disappeared.

From the second day after administration of the oil, dermatitis, characterized by local erythema appeared in the perianal area in four mice in group no. 4 and in all the mice in the last two groups. This decreased until day 14 when the animals were sacrificed.

Deaths were recorded from the second to the fifth day after administration of the oil. The autopsy of each showed no visceral damage. The histopathological analysis of the intestine showed atrophy and hyperplasia of intestinal villi in a dose-dependent manner, presenting mild atrophy and hyperplasia in group no. 4, moderate for group no. 5 and group 6. In samples from the perianal area (section proximal to the tail), mild dermal lesion was observed, with focal chronic prolymphocytic dermatitis in histology, and dose dependent from the third group onward, being moderate dermatitis and with infiltration of fat in groups 5 and 6.

LD_50_ was calculated by using Probits. For *P. volubilis* oil (density: 0.92551 g/mL), it was 63,603 mg/kg (63.6 g/kg), which, according to the criteria of Williams et al. (Annex I) (19) and the European Community standards for the classification of Acute Oral Toxicity, is considered NON-TOXIC.

In the case of *P. huayllabambana*, the following were obtained:

As in the case of the oil extracted from *P. volubilis*, in that of *P. huayllabambana* (**Table 6**), it was observed that there was mortality from group no. 4 (52.5 mL/kg). The animals also showed sweating from group no. 3 onward, ranging from mild sweating to moderate sweating, in a dose-dependent manner. On the second day after the administration of the oil, sweating decreased noticeably in the animals of group nos. 3 and 4 (30.2 and 52.5 mL/kg), whereas in the animals of group nos. 5 and 6 (91.2 and 150 mL/kg), sweating decreased from the fourth day. This decrease gradually subsided in the following days until it completely disappeared on the third day in group nos. 3 and 4, and on the seventh day in group nos. 5 and 6.

**Table 6:** Mortality data of mice treated with *P. huayllabambana* 7 days after treatment.

Group	Number animals	Average weight of mice (g)	Doses (mL/kg)	Managed volume (mL)	No. of deaths	No. of living
N°1	6	22.5	10	0.23	0	6
N°2	6	23.67	17.378	0.41	0	6
N°3	6	24.33	30.1995	0.74	0	6
N°4	6	25	52.4807	1.31	4	2
N°5	6	26.17	91.2011	2.39	3	3
N°6	6	30.67	150	4.60	4	2
Cooking oil	6	30.5	150	4.58	0	6

On the second day after administration of the oil, the appearance of dermatitis was observed in a similar way as in *P. volubilis*.

As in *P. volubilis*, the death of the animals was recorded from the fourth group, after administration of the oil. The necropsy performed on each of them also did not show any type of damage compared with the negative control.

In the histopathological analysis, the same results were obtained as in the case of *P. volubilis*.

The LD50 for Sacha Inchi oil produced from *P. huayllabambana*, for the density of the oil 0.92713 g/mL, was 74,638 mg/kg (74.6 g/kg), which, according to the criteria of Williams et al. (Annex I) (19), and the European Community standards for the classification of Acute Oral Toxicity, is considered NON-TOXIC.

### 3.3 Evaluation of cytotoxicity (IC_50_)

To find the IC_50_ for both oils, serial dilutions of 1 in 2 (volume/volume) were used, from 100% (which was pure Sacha Inchi oil) to 3.13%. The diluent was the RPMI + cyclodextrin complex.

From the optical densities obtained, the percentages of cellular toxicity were found for both oils, taking the cellular activity of the negative control (RPMI+ β-cyclodextrin) as 100%.

According to the percent cellular toxicity data found (**Table 7**), the GraphPad program calculated the following:

- For *P. volubilis*, an IC_50_ for 48 h was 48,561 mg/mL, while 50,468 mg/mL for 72 h.
- For *P. huayllabambana*, an IC_50_ for 48 h of 50,695 mg/mL, while 67,179 mg/mL for 72 h.

**Table 7:** Percentage of cellular activity for both oils.

% of activity	48 h		72 h	
	*P. volubilis*	*P. huayllabambana*	*P. volubilis*	*P. huayllabambana*
Negative control	100.00	100.00	100.00	100.00
100%	53.03	144.27	56.08	164.26
50%	129.05	148.00	131.93	169.43
25%	101.56	99.32	103.21	122.99
12.50%	84.21	109.00	103.69	134.41
6.25%	93.05	118.00	84.41	123.28
3.13%	76.59	83.42	96.13	122.92

Thus, because the IC_50_ doses found in this trial are impossible to achieve in cellular (tissue) conditions, it can be said that Sacha Inchi Oil, obtained from both seeds (*P. volubilis* and *P. huayllabambana*), is not cytotoxic. These data make Sacha Inchi oils very safe compounds.

### 3.4 Evaluation of the anti-inflammatory effect

The plantar edema model, developed by Sugishita et al. (18), proposes the induction of inflammation through λ-carrageenan.

Four experimental groups were established for each oil, in addition to three control groups (negative, positive, and anti-inflammatory), each with eight individuals. The statistical calculations were evaluating the data obtained 2 h after the induction. The statistical analysis were performed using the Mann–Whitney test or Wilcoxon rank sum of the GraphPad program.

In this way, for *P. volubilis* the results are shown in **Table 8**.

**Table 8:** Difference in inflammation (stimulated paw minus control paw) 2 h after injection with λ-carrageenan in mice treated with *P. volubilis*.

Individuals	Negative control	Positive control	Anti-inflamm control	5 mL/kg	10 mL/kg	20 mL/kg	40 mL/kg
N°1	0.10	1.01	0.73	1.11	0.79	0.52	0.61
N°2	0.08	1.41	0.40	1.09	0.93	0.85	0.90
N°3	0.00	1.29	0.71	1.04	0.99	0.44	0.80
N°4	0.04	1.41	0.57	1.02	1.11	0.61	0.22
N°5	0.05	1.02	0.46	0.90	0.98	0.79	0.77
N°6	0.03	1.00	0.29	1.34	1.02	0.80	0.41
N°7	-0.01	1.24	0.16	0.89	1.37	1.03	1.07
N°8	0.27	1.39	0.64	1.28	0.71	0.65	0.60
Average	**0.071**	**1.221**	**0.495**	**1.083**	**0.987**	**0.711**	**0.672**

Average (bold) in 8 individual mice in difference in inflammation. Data obained in millimeters (mm).

**Figure 4:**
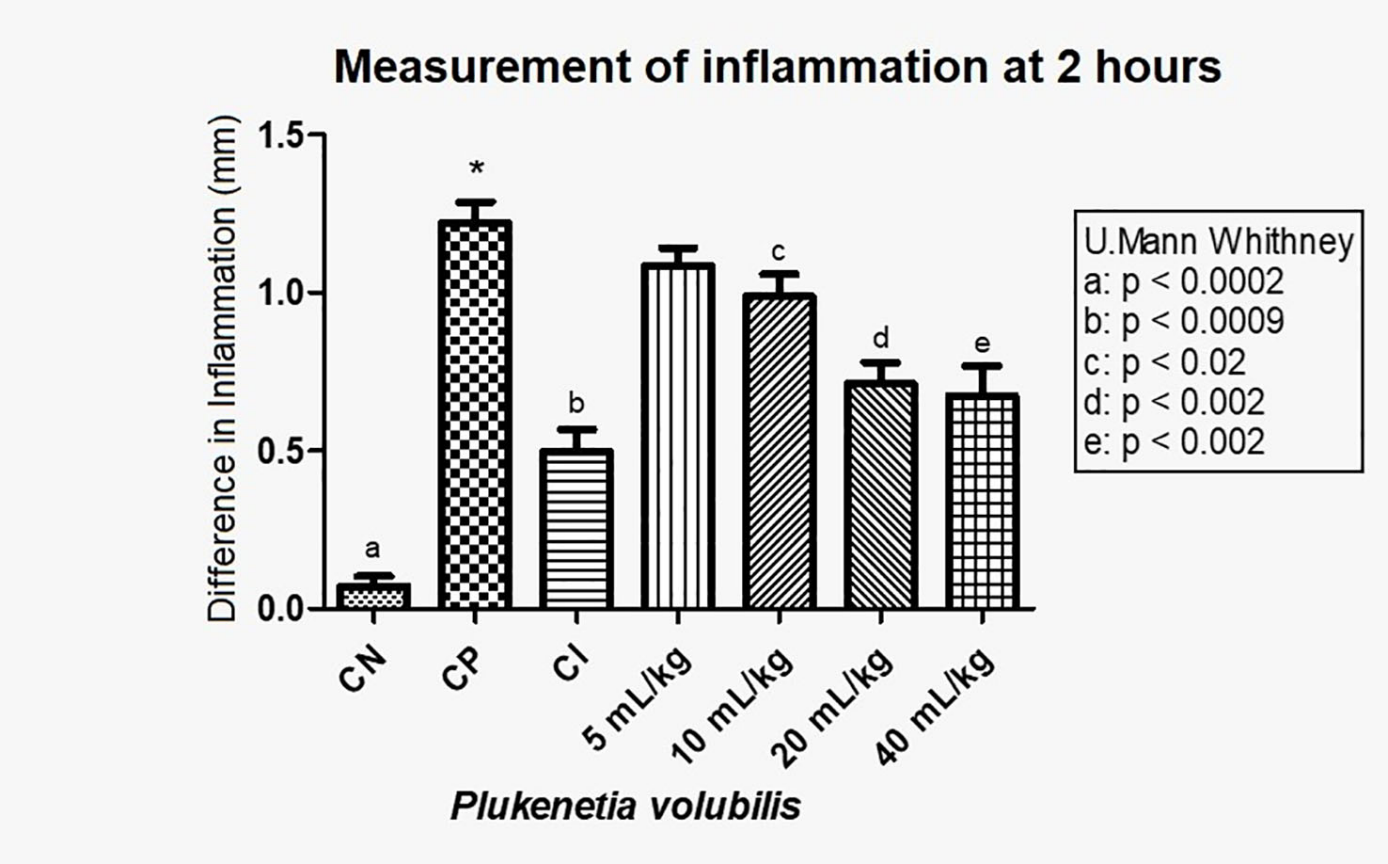
Difference in inflammation (stimulated paw minus control paw) 2 h after injection with λ-carrageenan in mice treated with *P. volubilis.* CN, negative control; CP, positive control; CI, anti-inflammatory control (indomethacin). a, b, c, d, and e denote a significant difference with respect to the positive control (*).

In **Figure 4**, with the averages of the measurements 2 h after induction of inflammation, a statistically significant decrease in inflammation (p <0.0009) can be observed in a dose-dependent manner.

**Figure 5:**
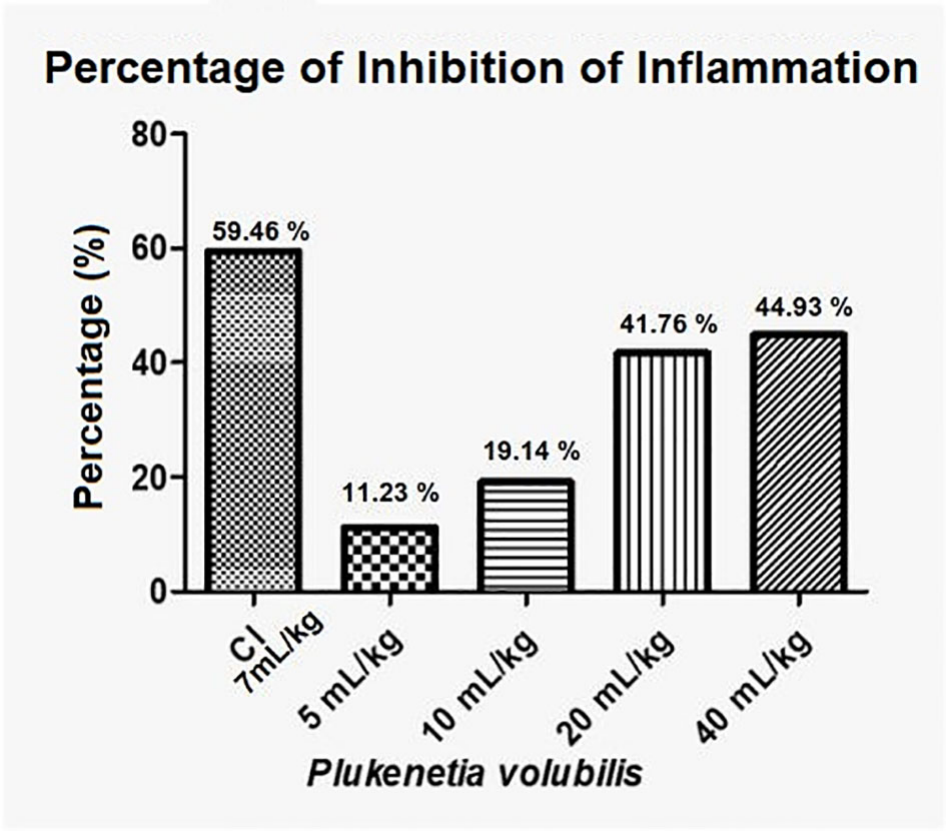
Percentage inhibition of inflammation for *P. volubilis.* IC, anti-inflammatory control (indomethacin).

**Figure 5** show that *P. volubilis*, at its highest dose (40 mL/kg), presents an inhibition percentage of 44.9%, still below the anti-inflammatory control (indomethacin).

For *P. huayllabambana*, we show results in **Table 9**:

**Table 9:** Difference in inflammation (stimulated paw minus control paw) 2 h after injection with λ-carrageenan in mice treated with *P. huayllabambana*.

Groups Individuals	Negative control	Positive control	Anti-inflammation control	5 mL/kg	10 mL/kg	20 mL/kg	40 mL/kg
**N°1**	0.10	1.01	0.73	1.06	0.10	0.80	0.51
**N°2**	0.08	1.41	0.40	1.04	0.79	0.85	0.63
**N°3**	0.00	1.29	0.71	0.85	0.70	0.83	0.52
**N°4**	0.04	1.41	0.57	0.80	1.28	0.91	0.55
**N°5**	0.05	1.02	0.46	1.29	0.89	0.90	1.11
**N°6**	0.03	1.00	0.29	0.92	0.88	0.96	0.10
**N°7**	-0.01	1.24	0.16	0.43	0.89	0.68	0.26
**N°8**	0.27	1.39	0.64	0.90	1.26	0.48	0.03
**Average**	**0.071**	**1.221**	**0.495**	**0.911**	**0.836**	**0.801**	**0.463**

Average (bold) in 8 individual mice in difference in inflammation. Data obained in millimeters (mm).

**Figure 6:**
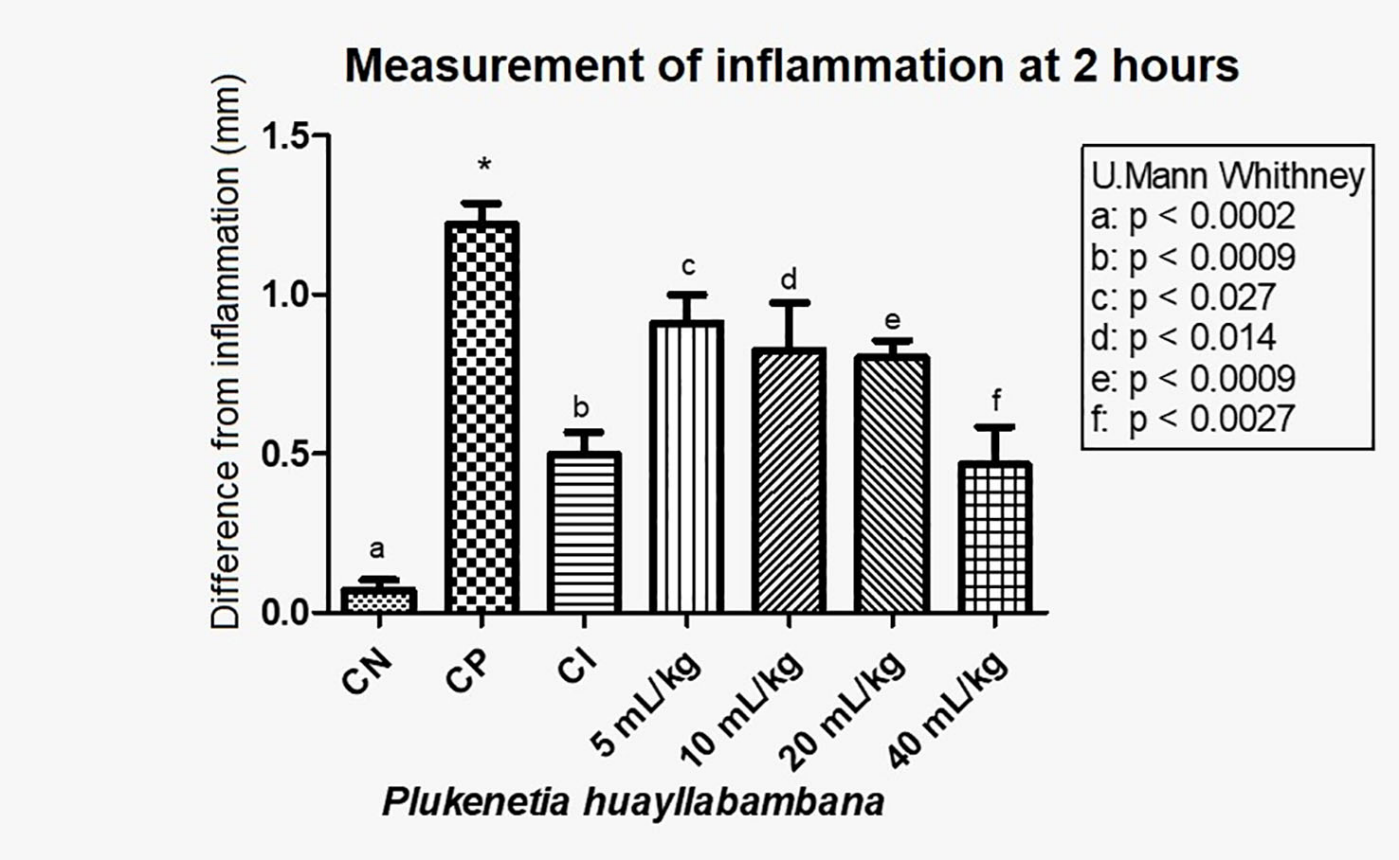
Difference in inflammation (stimulated paw – control paw) 2 h after injection with λ-carrageenan in mice treated with *P. huayllabambana.* CN, negative control; CP, positive control; CI, anti-inflammatory control (indomethacin). (a–f) denote a significant difference with respect to the positive control (*).

In **Figure 6**, with the averages of the measurements 2 h after induction of inflammation, a statistically significant decrease in inflammation was observed in a dose-dependent manner. A significant difference was found (see **Figure 6** for values) between the positive control and the anti-inflammatory control (indomethacin 7 mg/kg), as well as between the positive control and doses 5, 10, 20, and 40 mL/kg. Even for the 40-mL/kg dose, the level of inflammation was the same as with indomethacin (p>0.05)

These results indicate that Sacha Inchi oil produced from *P. huayllabambana* has an anti-inflammatory effect, which is even greater than the anti-inflammatory effect of *P. volubilis*, since a decrease in inflammation can be observed from the lowest dose (5 mL/kg).

**Figure 7:**
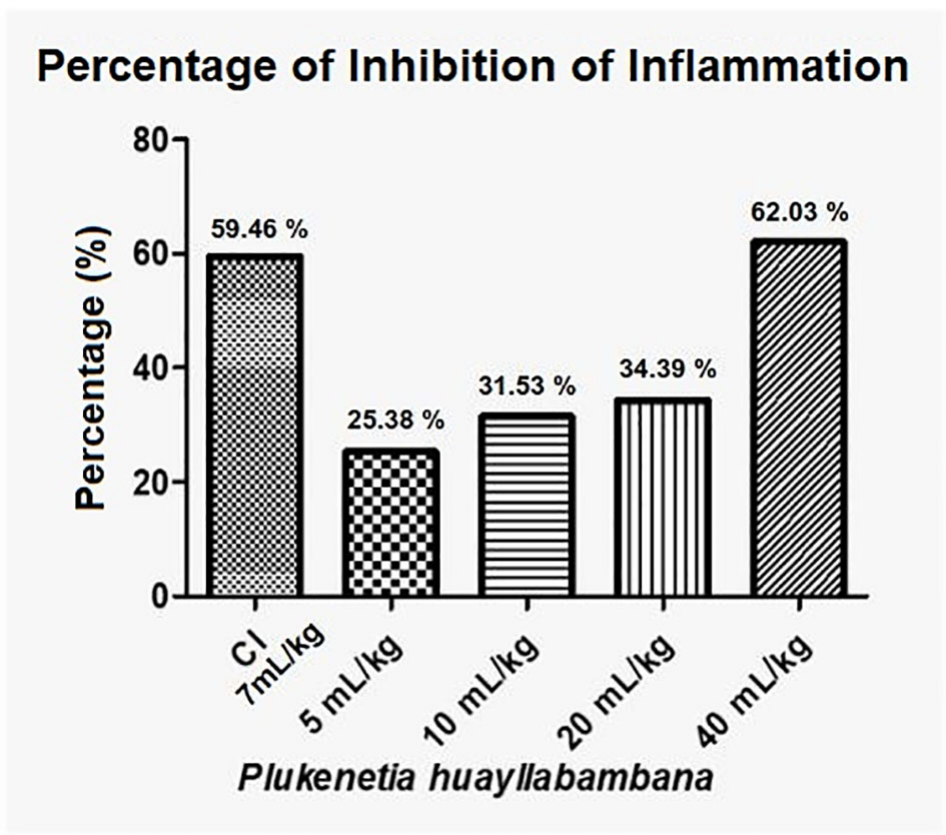
Anti-inflammatory percentage or inflammation inhibition percentage for *P. huayllabambana.* IC, anti-inflammatory control (indomethacin).

Regarding the inhibition of inflammation, *P. huayllabambana*, in its highest dose (40 mL/kg), presents an inhibition percentage of 62% (**Figure 7**), which is even higher than the inhibition percentage of the anti-inflammatory control (indomethacin), this being a highly potent synthetic anti-inflammatory and was administered at a high dose.

## 4 Discussion

Omega fatty acids, particularly ω-3 and its relationship with inflammation, have been widely studied in recent years, revealing a strong association between the consumption of ω-3 and the inhibition of the inflammatory process in patients with autoimmune diseases or chronic inflammation. ^5.9-16.^

Given the popular information about the high ω-3 content in this oil, the motivation arose to check the lipid profile of the oils commercially available in Peru and evaluate the potential anti-inflammatory capacity of the oil. For this, an *in vivo* inflammation model was used, but previously, we determined the LD_50_ for both oils, to ensure that anti-inflammatory doses were within the permissible range.

The results demonstrate in an *in vivo* model that Sacha Inchi oil has anti-inflammatory capacity in its two varieties, both that extracted from *P. volubilis* seeds and from *P. huayllabambana*, even having a higher percentage of inhibition of inflammation than the anti-inflammatory control (indomethacin). Of both oils, *P. huayllabambana* was the one that presented a greater effect.

This difference in anti-inflammatory activity between one variety of Sacha Inchi and another may be based on the different composition of fatty acids found in each one (*P. huayllabambana* 53.20% α-linolenic acid [ω-3] and 29.17% linoleic acid [ω-6], while 46.65% ω-3 acids and 35.53% ω-6).

A very early report by Hamaker et al. (22) evaluated the fatty acid composition of Sacha Inchi (*P. volubilis*) oil and found 45.2% α-linolenic fatty acid and 36.80% linoleic acid. Likewise, Gorriti et al. (23) found 47.06% ω-3 and 36.19% ω-6. Both results are quite close to those found in the present investigation.

In the acute toxicity by the LD_50_ test of both oils, the LD_50_ for *P. volubilis* was found to be 63,603 mg/kg (63.6 g/kg), whereas for *P. huayllabambana*, it was 74,638 mg/kg (74.6 g/kg), demonstrating that both oils are relatively harmless according to the classification of Williams et al. (19), which corresponds to the best rating of this classification; that is, they are non-toxic products.

Córdova et al. (24) showed an LD_50_ in rats of 103.4 g/kg for *P. volubilis*, whereas Gorriti et al. (23) had an LD_50_ value in mice of 37 g/kg, for the same variety of oil. These differences in LD_50_ may be due to several reasons, perhaps the most obvious being the fact that they are different products, coming from a different selection of seeds. In the present work, they came from the lowland forest of the department of San Martín and the province of Rodríguez de Mendoza in the department of Amazonas, whereas the others do not specify exactly. Additionally, a diverse extraction protocol (in this study was cold pressed). It also differs in the use of different experimental animals (mice and rats of strains different). Therefore, it is expected that there will be a different concentration of active metabolites and different animal responses.

In the cytotoxicity test (IC_50_), both oils showed an IC_50_ with values so high that it would be impossible to reach them under normal cellular conditions. Thus, it can be said that Sacha Inchi oil is not cytotoxic. Even more, the cell activity values (**Table 7**) found for the highest concentrations (100, 50, and 25% for *P. huayllabambana* and 50 and 25% for *P. volubilis*) are values ​​higher than those of the negative control, suggesting that Sacha Inchi oil could induce an increase in cell viability.

In the test of the anti-inflammatory effect of plantar edema, the data 2 h after inflammation show that both oils have a statistically significant anti-inflammatory effect (**Figures 4**, **6**). For *P. volubilis*, the significant difference was from 10 mL/kg (p<0.02), whereas for *P. huayllabambana*, it was from 5 mL/kg (p<0.027). A significant difference was also found in the percentage of inflammation inhibition (**Figures 5**, **7**) for both oils: *P. volubilis* obtained 44.93% for its highest dose (40 mL/kg), and *P. huayllabambana* obtained 62.03% for the same dose. Even for *P. huayllabambana*, the percentage of inflammation inhibition was higher than that of the anti-inflammatory control (indomethacin) (59.46%).

The concentration of fatty acids was different in each variety. It is likely that the difference in the anti-inflammatory effect between both varieties is due to the different concentration of ω-3, which is consistent with previous research in which it was shown that the greater the consumption of ω-3, the greater the anti-inflammatory effect (5–9). Our findings show that Sacha Inchi oil, particularly that extracted from *P. huayllabambana*, exhibits a significant anti-inflammatory effect, in the plantar edema model. This difference in efficacy appears to be related to its higher ALA and ω-3 contents. Omega-3 acids are known to modulate inflammation by inhibiting the production of pro-inflammatory eicosanoids (such as prostaglandins and leukotrienes) derived from arachidonic acid, and by promoting the synthesis of pro-resolving lipid mediators such as resolvins and protectins. Additionally, the abundance of ω-3 fatty acids may influence cell membrane fluidity, which in turn modulates surface receptor activity and intracellular signaling pathways involved in the inflammatory response.

Previous studies on Sacha Inchi have determined its physicochemical properties, its fatty acid content, and its protein profile; some have even demonstrated its lipid-lowering capacity in animal models, and in humans (2, 3, 16, 22–25). However, it is the first time that the anti-inflammatory effect of Sacha Inchi oil has been demonstrated *in vivo*.

From a translational perspective, the findings in this animal model lay an important foundation for exploring the use of Sacha Inchi oil in humans. However, the doses used (5–40 mL/kg) may not be directly extrapolable to humans without proper conversion and evaluation of bioavailability. For instance, using the FDA-recommended interspecies conversion factor (from rats to humans), an effective dose of 10 mL/kg in rats would correspond approximately to 1.62 mL/kg in humans—approximately 97 mL/day for a 60-kg adult. This amount exceeds what would be considered a typical dietary intake, suggesting that formulation studies are needed to concentrate active fatty acids, improve oxidative stability, and enable safe and effective dosing in humans. Moreover, although the oil was found to be non-cytotoxic and relatively harmless in acute toxicity tests (LD50 > 63 g/kg), further studies on subchronic toxicity and the bioavailability of active metabolites (e.g., resolvins, protectins) are essential next steps.

In summary, this study not only scientifically confirms the anti-inflammatory capacity of Sacha Inchi oils *in vivo* but also suggests a plausible biochemical basis related to its ω-3 content, which can serve as a basis for the development of subsequent studies in animals and eventually for the design of studies of anti-inflammatory activity in humans, as well as develop optimized formulations, subchronic toxicity, and bioavailability of active biomarkers metabolites in humans (e.g., resolvins, protectins).

## Data Availability

The raw data supporting the conclusions of this article will be made available by the authors, without undue reservation.
